# Meta-analysis of the prognostic value of lncRNA ZFAS1 in patients with solid tumors

**DOI:** 10.18632/oncotarget.19566

**Published:** 2017-07-26

**Authors:** Wei Song, Chuan Tian, Run-Jin Zhang, Shu-Bing Zou, Kai Wang

**Affiliations:** ^1^ Department of Hepatobiliary Surgery, The Second Affiliated Hospital of Nanchang University, Nanchang, China; ^2^ Department of Nuclear Medicine, Guizhou Provincial People's Hospital, Guiyang, China

**Keywords:** ZFAS1, long noncoding RNA, cancer, prognosis, meta-analysis

## Abstract

Expression of ZFAS1, a newly identified long noncoding RNA (lncRNA), is dysregulated in several types of cancer. Here we assessed the prognostic value of ZFAS1 in solid tumors. A comprehensive literature search was performed by screening the PubMed, EMBASE, MEDLINE, Cochrane Library, CNKI, and Wanfang databases. A total of 874 patients from 10 studies were included. The pooled analysis demonstrated that patients with high ZFAS1 expression had a significantly shorter overall survival (OS) (HR, 1.58; 95% CI, 1.28–1.97; *P* < 0.001) and recurrence-free survival (RFS) (HR, 1.90; 95% CI, 1.29–2.79; *P* = 0.001). Moreover, elevated ZFAS1 expression correlated with tumor size, tumor-node-metastasis (TNM) stage, and lymph node metastasis (LNM). These results demonstrate that increased ZFAS1 expression correlates with a poor prognosis in cancer patients, which suggests ZFAS1 might be useful as a potential prognostic biomarker in patients with solid tumors.

## INTRODUCTION

Cancer is a major cause of morbidity and mortality worldwide [1, 2]. Despite significant advances in medical care, the prognosis of cancer is still extremely poor. Therefore, it is vital to develop specific and sensitive biomarkers for early diagnosis and more accurate cancer prognosis.

Long noncoding RNAs (lncRNAs) are non-protein-coding RNAs that are greater than 200 nucleotides [3]. Despite the outdated opinion that lncRNAs simply represent a transcriptional noise [4], emerging evidence suggests that lncRNAs play a pivotal role in the development and progression of cancer [5, 6]. LncRNAs regulate a variety of biological processes, including gene regulation at the chromatin, transcriptional, and post-transcriptional levels [7, 8]. In fact, lncRNAs have been recognized as hallmarks of the onset and development of various types of cancer [9–12].

Zinc finger antisense 1 (ZFAS1) is a transcript antisense to the 5’ end of the protein-coding gene Znfx1, and hosts three C/D box snoRNAs (SNORDs): Snord12, Snord12b, and Snord12c. SnoRNAs consist of a group of non-coding RNAs with a length of 60 to 150 nt. Structurally, snoRNAs are divided into C/D-box snoRNAs and H/ACA-box snoRNAs (SNORAs). SNORDs mainly direct ribosome RNA site-specific methylation. SNORDs are dysfunctional in various types of cancer, and this dysfunction may be associated with the development and progression of various malignancies [13–15]. In vertebrates, most snoRNAs are intron-encoded. Although some snoRNAs genes can encode proteins, most of them are non-coding RNAs [16]. One subgroup of lncRNAs is composed of the host gene of snoRNA, ZFAS1 [17, 18]. A study by Askarian-Amiri et al. has indicated that ZFAS1 is downregulated in human breast cancer [18]. Knockdown of ZFAS1 in mammary epithelial cells increases their proliferation and differentiation, suggesting that ZFAS1 may serve as a tumor suppressor gene. However, recent studies have shown that ZFAS1 is upregulated in multiple types of tumors, including hepatocellular carcinoma, gastric cancer, and colorectal cancer [19–22], suggesting that ZFAS1 may serve as a proto-oncogene, and a prognostic biomarker in cancer. Furthermore, elevated expression of ZFAS1 has been associated with worse overall survival (OS) and recurrence-free survival (RFS) rates in cancer patients [23, 24].

Nevertheless, the prognostic value of ZFAS1 in cancer has not yet been fully elucidated, and systematic studies are lacking. Therefore, we performed a meta-analysis to assess the prognostic value of ZFAS1, and examine its clinicopathological features in patients with various solid tumors.

## MATERIALS AND METHODS

### Search strategies

The present study was performed in accordance with the meta-analyses guidelines [25]. We searched PubMed, EMBASE, MEDLINE, Cochrane Library, CNKI, and Wanfang databases from inception up to June 2017. Search terms included: “long noncoding RNA”, or “lncRNA”, or “ZFAS1”, or “zinc finger antisense 1”, or “ZNFX1 antisense RNA 1”, or “SNORD12”, or “small nucleolar RNA, C/D box 12”, “cancer”, or “tumor”, or “carcinoma”, or “neoplasms”, “prognostic”, or “prognosis”, or “survival”, or “mortality”, or “recurrence”, or “outcome”. In addition, the references of eligible studies, relevant systematic reviews, and meta-analyses in this field were manually retrieved.

### Study selection

The criteria for inclusion were as follows: (1) studies of patients with any type of cancer; (2) assessing the association of ZFAS1 with OS and/or RFS or clinicopathological features; (3) reporting a sufficient information to estimate the hazard ratio (HR) and 95% confidence interval (CI). The exclusion criteria were: (1) reviews, letters, case-reports, and conference abstracts; (2) lacking essential information for calculating an HR and 95% CI; and (3) overlapping or duplicate data.

### Data extraction

Two investigators reviewed and extracted the data independently. The following information was collected: author's name, year of publication, country, number of patients, patient characteristics (sex, tumor type), duration of follow-up, ZFAS1 expression, cut-off values, detection method, clinicopathological features (LNM, vascular invasion, and tumor stage), and outcome measures (OS and RFS). HRs were extracted from multivariate or univariate analyses or estimated from Kaplan-Meier survival curves [26]. Any disagreement was resolved by a third reviewer.

The Newcastle-Ottawa Scale (NOS) was used to assess the quality of each study [27]. This scale mainly includes subject selection, comparability of groups, and clinical outcome three categories. A total of nine items were extracted and each item scored 1. The total scores ranged from 0 to 9. If a score was ≥ 6, the study was considered as high quality.

### Data synthesis and statistical analyses

The meta-analysis was conducted using RevMan 5.3 software (Cochrane Collaboration, Copenhagen, Denmark). Heterogeneity of the HR of each study was quantified using the chi-squared based Q-statistic test. The assumption of homogeneity was considered invalid for I^2^ > 50% and *P* < 0.10. When there was no statistically significant heterogeneity, we used the fixed-effects model for pooling the results; otherwise, the random-effects model was applied. HRs and 95% CIs were searched in the original articles or extrapolated using methods described by Tierney and Parmar [26, 28]. The log HR and standard error (SE) were used for aggregation of the survival results [28]. The associations between ZFAS1 and clinicopathologic features were expressed as odds ratios (ORs) and their 95 % CIs. Publication bias was evaluated using funnel plots and with the Begg and Egger tests [29, 30]. *P* < 0.05 was defined as statistically significant.

## RESULTS

### Search results

**Figure 1:**
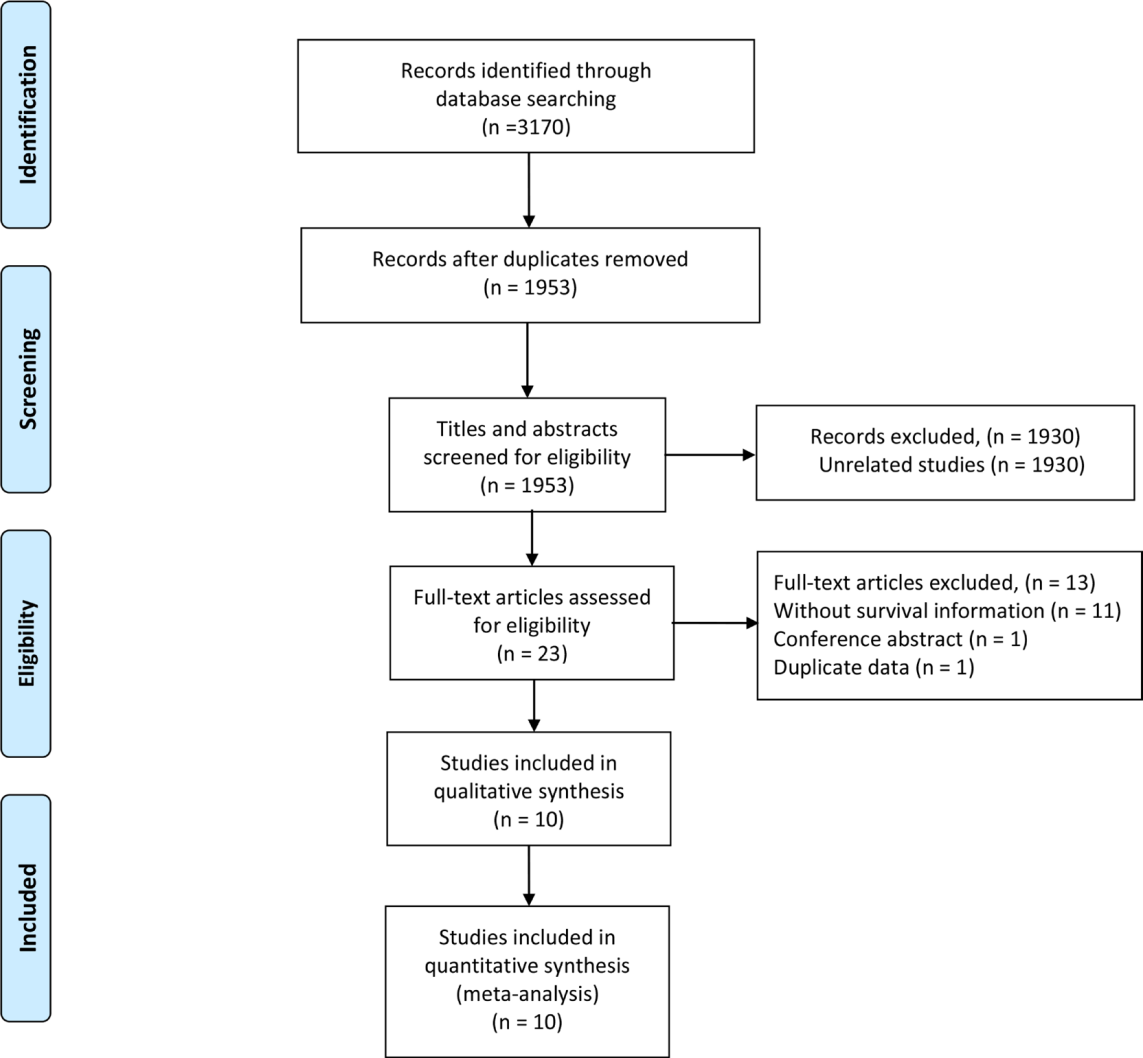
Flow diagram of the study selection process

Our search strategy yielded 3,170 potentially relevant articles. After excluding duplicate articles, 1,953 potentially eligible studies were selected. 1,930 studies were excluded after screening titles and abstracts. 23 relevant studies were selected for further evaluation; 13 studies were excluded after reviewing the full article. Thus, 10 studies, comprising a total of 874 patients, were included in the quantitative synthesis [19, 21, 23, 24, 31–36]. The selection process is shown in Figure 1.

### Characteristics of the included studies

All studies were published in 2015 or later, and conducted in China. The number of patients in each study varied from 54 to 173. Various cancers were recorded in our study, including hepatocellular carcinoma (HCC), gastric cancer (GC), colorectal cancer (CRC), non-small cell lung cancer (NSCLC), ovarian cancer (OC), colonic cancer (CC), and glioma. There were eight studies for OS, three for RFS, seven for lymph node metastasis (LNM), three for vascular invasion, and nine for cancer stage enrolled in the database-based analysis. HRs and 95% CIs in three studies were extracted directly. HR values in five studies were estimated by Kaplan-Meier survival curves. In methodological quality of studies, the overall NOS scores ranged from 6 to 8. Detailed patient characteristics are shown in Table 1.

**Table 1:** Characteristics of the studies included in the meta-analysis

Author	Year	Country	Cancer type	No. of patients	Tumor Stage	Cut-off value	Survival endpoint	HR (95% CI)	Method	NOS score
Li	2015	China	HCC	113	Mixed	Median	OS/RFS	OS: 1.58 (0.75–3.31) RFS: 1.76 (1.05–2.94)	qRT-PCR	8
Fang	2016	China	CC	73	Mixed	10.84	OS	1.27 (0.88–1.83)	qRT-PCR	8
Nie	2016	China	GC	54	Mixed	Median	OS/RFS	OS: 2.14 (0.56–8.25) RFS: 2.03 (0.82–5.02)	qRT-PCR	8
Tian	2016	China	NSCLC	173	Mixed	NA	OS	1.83 (1.04–3.83)	qRT-PCR	7
Wang	2016	China	CRC	159	Mixed	Median	OS/RFS	OS: 1.88 (1.01–3.53) RFS: 2.13 (1.02–4.55)	qRT-PCR	8
Wu	2016	China	CRC	67	Mixed	Median	NA	NA	qRT-PCR	6
Gao	2017	China	glioma	46	NA	NA	OS	2.15 (0.79–5.88)	qRT-PCR	6
Xia	2017	China	OC	60	Mixed	NA	OS	1.39 (0.69–2.79)	qRT-PCR	7
Pan	2017	China	GC	60	Mixed	2.0	NA	NA	qRT-PCR	6
Lv	2017	China	glioma	69	Mixed	Median	OS	1.92 (1.06–3.47)	qRT-PCR	7

Abbreviations: GC, gastric cancer; HCC, hepatocellular carcinoma; PC, pancreatic cancer; OC,

ovarian cancer; CC, colonic cancer; OS, overall survival; RFS, recurrence-free survival; qRT-PCR,

quantitative real-time PCR; NA, not available.

### Meta-analysis: ZFAS1 expression, OS, and RFS in cancer

**Figure 2:**
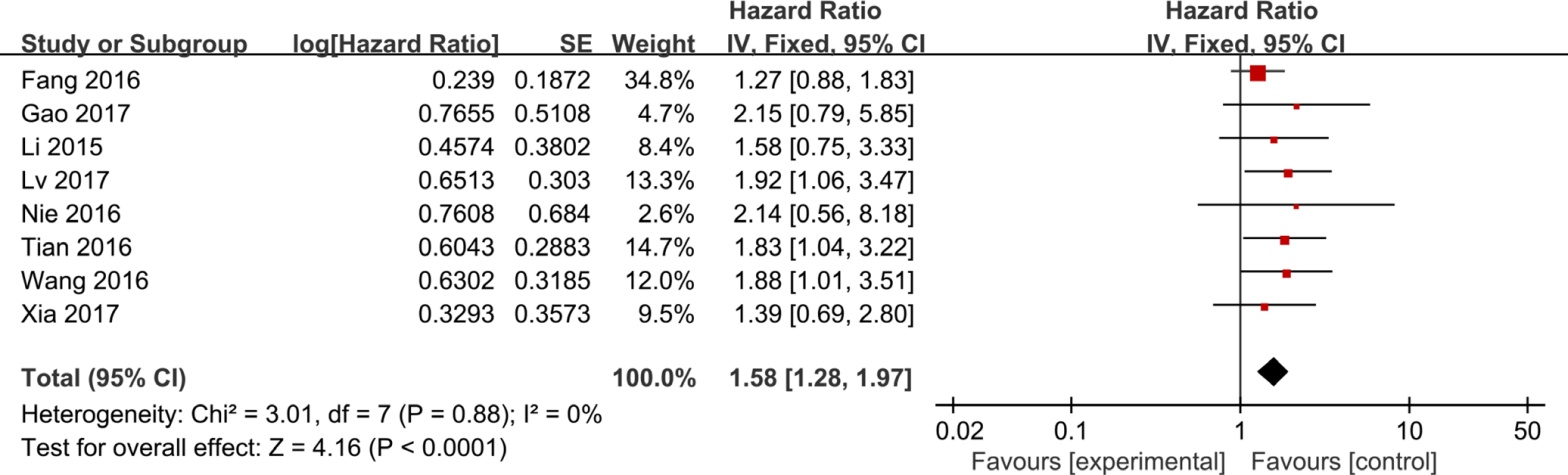
Forest plots for the association between ZFAS1 expression and OS

**Figure 3:**
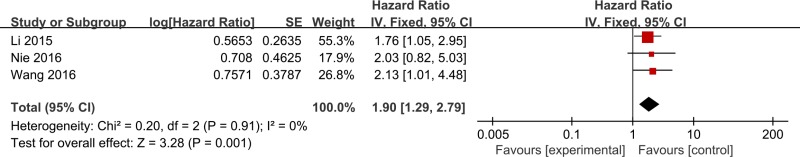
Forest plots for the association between ZFAS1 expression and RFS

Eight studies reported data on ZFAS1 expression and OS. The results showed that elevated ZFAS1 expression was associated with a poor OS (HR, 1.58; 95% CI, 1.28–1.97; *P* < 0.001; Figure 2). There was no significant heterogeneity among studies (I^2^ = 0%; *P* = 0.88); thus, the fixed-effects model was used. A meta-analysis of HRs for RFS was performed on three studies, and the negative prognostic effect of a high ZFAS1 expression on RFS was again observed (HR, 1.90; 95% CI, 1.29–2.79; *P* = 0.001; Figure 3) using the fixed-effects model (heterogeneity test: I^2^ = 0%; *P* = 0.91).

### ZFAS1 expression and clinicopathological parameters in cancer

In the meta-analysis, we evaluated the impact of ZFAS1 expression on 7 clinical features in cancer patients. The pooled analysis demonstrated that elevated ZFAS1 expression correlated with tumor size (> 5 cm vs. < 5 cm; OR = 1.42, 95% CI: 1.03–1.94, *P* = 0.03), TNM stage (III-IV vs. I-II; OR = 2.31, 95% CI: 1.35–3.95, *P* = 0.002), and LNM (pos vs. neg; OR = 2.20, 95% CI: 1.15–4.18, *P* = 0.02). No significant association was found with gender (male vs. female), age (≥ median vs. < median), differentiation (low vs. moderate/high), and vascular invasion (pos vs. neg). The correlation between ZFAS1 expression and clinicopathological parameters is shown in Table 2.

**Table 2:** Meta-analysis of the association between AFAS1 and clinicopathological features

Characteristics	No. of studies	No. of patients	OR (95% CI)	*p*	Heterogeneity	
					I^2^ (%)	P_h_
Gender (male vs. female)	8	768	1.01 (0.76–1.35)	0.94	6	0.39
Age (≥ median vs. < median)	10	874	0.99 (0.75–1.31)	0.95	4	0.40
Differentiation (low vs. moderate/high)	6	586	1.39 (0.74–2.61)	0.30	60	0.03
Tumor size (> 5 cm vs. < 5 cm)	6	626	1.42 (1.03–1.94)	0.03	15	0.31
TNM stage (III–IV vs. I–II)	10	874	2.31 (1.35–3.95)	0.002	68	0.001
Lymph node metastasis (pos vs. neg)	7	646	2.20 (1.15–4.18)	0.02	70	0.003
Vascular invasion (pos vs. neg)	3	246	1.80 (0.79–4.10)	0.16	53	0.12

**Figure 4:**
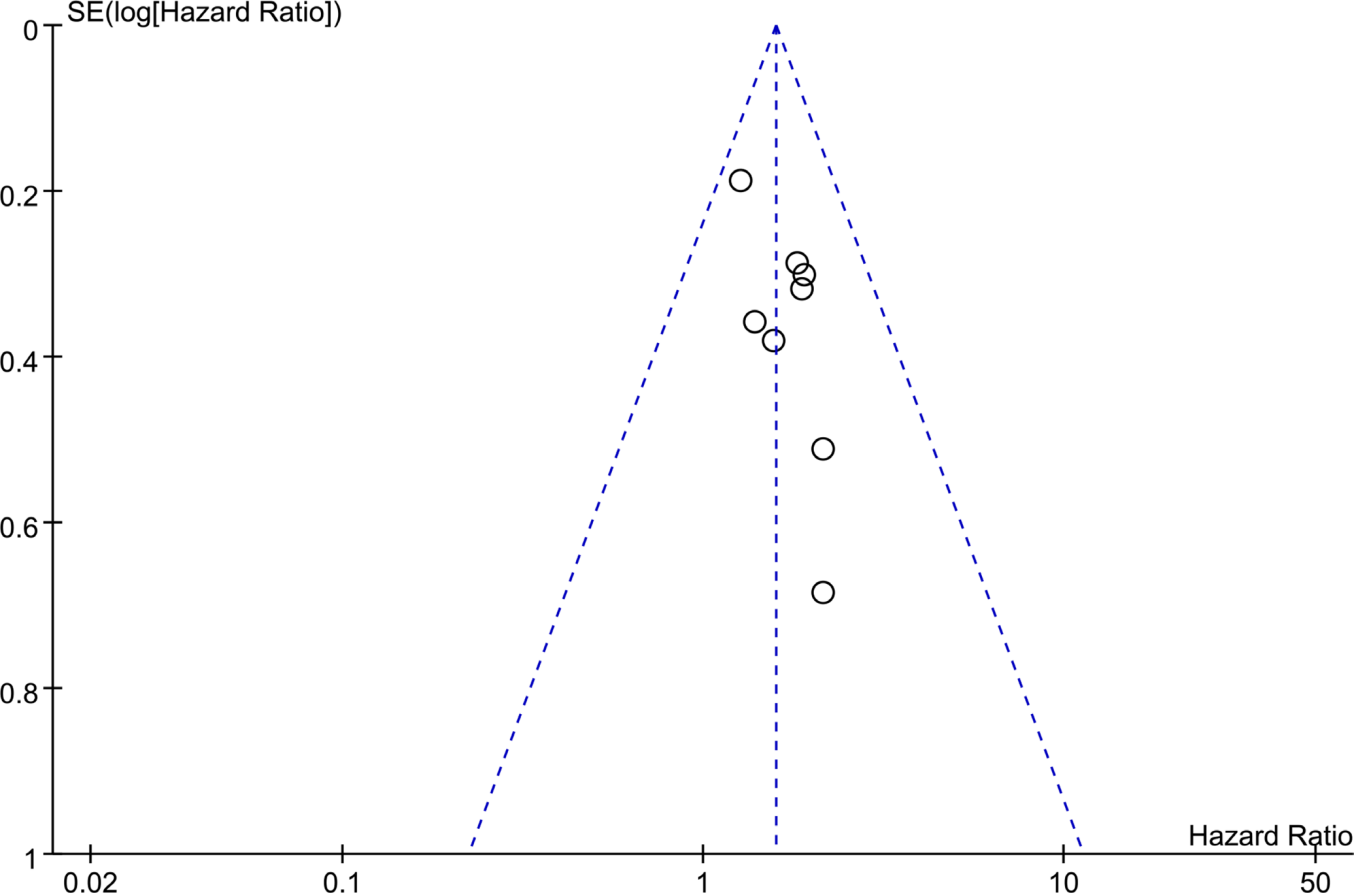
Funnel plot for the publication bias test of the included studies for ZFAS1 expression and OS

The Begg's funnel plot and Egger's test were performed to evaluate the publication bias. The shape of the funnel plot was asymmetric, suggesting a high risk of publication bias (Figure 4).

## DISCUSSION

Increased ZFAS1 expression has been associated with malignant progression, metastasis, and poor prognosis in several types of cancer [31–33]. In hepatocellular carcinoma, ZFAS1 expression was found increased in cancer tissues compared to normal tissues, and correlated with higher recurrence rates and shorter OS [19]. Similarly, elevated ZFAS1 expression was observed in colorectal cancer patients, and correlated with lymphatic invasion, advanced TNM stages, and poor survival. *In vitro*, ZFAS1 suppression decreased cell migration and invasive ability of colorectal cancer cells [21]. These studies have suggested that the ZFAS1 expression might serve as a promising prognostic and therapeutic target in cancer treatment.

To our knowledge, this is the first meta-analysis that evaluates the association of ZFAS1 expression with prognosis and clinicopathological features in various cancers. Our meta-analysis provides a strong evidence that high levels of ZFAS1 correlate with poor OS and RFS rates. Moreover, our results show that elevated ZFAS1 levels correlate with tumor size, TNM stage, and LNM.

The mechanisms responsible for the association between high ZFAS1 expression and poor survival in cancer patients remain unclear. However, several experimental studies have shown that ZFAS1 plays a pivotal role in tumor progression by regulating cell proliferation, invasion, apoptosis, and migration [23, 32, 37]. In gastric cancer, knockdown of ZFAS1 exerts tumor-suppressive functions through reducing cell proliferation and inducing cell apoptosis. The ZFAS1-mediated pro-oncogenic effect is partially through its epigenetic silencing of Kruppel-like factor 2 (KLF2) and Naked cuticle 2 (NKD2) expression by binding with polycomb repressive complex 2 (PRC2) and lysine-specific demethylase 1 [23]. Furthermore, *in vitro* studies have indicated that ZFAS1 enhances proliferation and invasion of colorectal cancer cells by interaction with CDK1/cyclin B1 complex and destabilization of p53 [22]. Moreover, ZFAS1 has been shown to function as an oncogene in HCC progression by sponging miR-150 and de-repressing its regulation of zinc finger E-box binding homeobox 1 protein, matrix metalloproteinase 14 (MMP14), and matrix metalloproteinase 16 (MMP16) expression [19].

The limitations of our study include the small sample size, and the fact that the cut-off value of ZFAS1 expression differed in each study. This might be a significant contributor to the substantial clinical heterogeneity. In addition, since the HRs and 95% CIs values were estimated from Kaplan-Meier survival curves, the prognostic value of ZFAS1 expression may be overestimated. The fact that all included studies were from China limits our conclusions for other ethnic populations. Furthermore, since our aim was to gain a general insight into the overall prognostic value of ZFAS1 expression in cancer patients, this study included different types of cancer. However, different cancers are likely to have different pathogenic mechanisms, and the same gene may play different roles in various cancers; this further affects the reliability of the results and their practical significance.

In summary, our meta-analysis demonstrates that elevated ZFAS1 expression correlates with poor prognosis in cancer patients, suggesting that ZFAS1 might serve as a potential molecular target for cancer prognosis.
